# Novel Telomere-Anchored PCR Approach for Studying Sexual Stage Telomeres in *Aspergillus nidulans*

**DOI:** 10.1371/journal.pone.0099491

**Published:** 2014-06-13

**Authors:** Nengding Wang, Saajidha Rizvydeen, Mithaq Vahedi, Daysi M. Vargas Gonzalez, Amanda L. Allred, Dustin W. Perry, Peter M. Mirabito, Karen E. Kirk

**Affiliations:** 1 Biology Department, Lake Forest College, Lake Forest, Illinois, United States of America; 2 Biology Department, University of Kentucky, Lexington, Kentucky, United States of America; University of North Carolina, United States of America

## Abstract

Telomere length varies between germline and somatic cells of the same organism, leading to the hypothesis that telomeres are lengthened during meiosis. However, little is known about the meiotic telomere length in many organisms. In the filamentous fungus *Aspergillus nidulans*, the telomere lengths in hyphae and asexual spores are invariant. No study using existing techniques has determined the telomere length of the sexual ascospores due to the relatively low abundance of pure meiotic cells in *A. nidulans* and the small quantity of DNA present. To address this, we developed a simple and sensitive PCR strategy to measure the telomere length of *A. nidulans* meiotic cells. This novel technique, termed “telomere-anchored PCR,” measures the length of the telomere on chromosome II-L using a small fraction of the DNA required for the traditional terminal restriction fragment (TRF) Southern analysis. Using this approach, we determined that the *A. nidulans* ascospore telomere length is virtually identical to telomeres of other cell types from this organism, approximately 110 bp, indicating that a surprisingly strict telomere length regulation exists in the major cell types of *A. nidulans*. When the hyphal telomeres were measured in a telomerase reverse transcriptase (TERT) knockout strain, small decreases in length were readily detected. Thus, this technique can detect telomeres in relatively rare cell types and is particularly sensitive in measuring exceptionally short telomeres. This rapid and inexpensive telomere-anchored PCR method potentially can be utilized in other filamentous fungi and types of organisms.

## Introduction

Telomeres, consisting of repetitive DNA sequences and associated proteins, are the protective caps at the termini of natural linear chromosomes. One of the important functions of telomeres is to overcome the end replication problem and maintain a stable genome as cells divide [1], [2]. The repetitive sequence differs in nucleotide composition, and the overall length of the telomeres varies widely between organisms. Telomere length variance also exists between different cell types within the same organism, which is particularly significant in germ cells [3], [4]. For example, human sperm cells possess longer telomeres (10–14 kb) than average somatic cells, which are several kilobases shorter in length [5]. Such findings have led to the hypothesis that telomeres are lengthened for the purpose of “resetting” during gametogenesis [6], which would replenish the telomere length that would otherwise naturally shrink during cell replication and organismal growth [7]. It has also been speculated that if telomere lengthening is dysfunctional during gametogenesis, premature decrease in female fertility might occur [8].

Telomere function is known to be conserved prior to meiosis: telomeres attach to the nuclear periphery and are involved transiently in forming the “bouquet” structure, hypothesized to contribute to chromosome alignment and to spindle pole body assembly in fission yeast cells [9], [10], [11], [12]. It is unknown whether organisms like fission yeast and other microbial eukaryotes normally have longer telomeres in meiosis compared to somatic cells, as this phenomenon has yet to be extensively studied in these organisms. In the cytoplasm of the single-celled ciliated protozoan *Tetrahymena thermophila*, the telomeres of the germline micronucleus are several fold longer than the telomeres of the somatic macronucleus [13]. It is not known, however, whether this difference is due to a requirement for longer telomeres during meiosis in the germline or whether it is due, for instance, to the extreme difference in size of the entire chromosomes between the micronucleus and macronucleus, which comprises mini-chromosomes.

Telomere biology has not been extensively studied in filamentous fungi [14]. Recently, the telomerase RNA sequence was identified in several Aspergilli [15] and in a range of other filamentous fungi [16], thus some molecular tools are now available. Moreover, *Aspergillus nidulans* provides a genetically tractable model system, in particular to study telomere length in a number of different cell types such as asexual conidia and sexual ascospores. *A. nidulans* has ascospores that are uniquely housed in a separate structure called the cleistothecium. One cleistothecium holds 10,000 or more ascospores [17], but unfortunately the external surfaces of cleistothecia are covered by conidia and other cell types. These cells are extremely labor intensive to remove, and thus pure ascospore DNA can be obtained only in small quantities. Consequently, meiotic telomere length cannot be determined by conventional means, and meiotic telomere length remained unknown in *A. nidulans*.

The overall telomere tract in *A. nidulans*, composed of TTAGGG repeats, is exceptionally uniform in size, ranging from 90–120 bp [18]. Furthermore, the telomere length is constant throughout vegetative development of the organism, even under different growth temperatures; this suggests stringent regulation [18]. In this report, the meiotic telomere length was analyzed to determine whether telomeres are universally longer in germ cells compared to somatic cells, presumably to aid in some aspect of chromosome alignment or bouquet formation. To test this hypothesis, a novel method to determine telomere length using small quantities of DNA was first developed.

In most organisms, telomere length can be determined by terminal restriction fragment (TRF) Southern blot analysis [19]. Such a method was employed successfully to detect telomere length in abundant, vegetative *A. nidulans* cell types [18], where microgram quantities of DNA can be obtained readily. Other telomere length measurement techniques, such as single telomere length analysis (STELA), are PCR based [20], [21] and are several orders of magnitude more sensitive to the quantities of DNA. However, one drawback to STELA is that it does not determine the terminal nucleotide at the G-rich 3′ overhang. To overcome the limitations posed by other methods, our laboratory developed and validated a novel “telomere-anchored PCR” in *A. nidulans*.

In this study, we demonstrate the efficacy of the first PCR method used in filamentous fungi and apply it to detect differences in telomere length of wild-type and TERT knockout cells in *A. nidulans*. Moreover, this method was also utilized to measure telomere length of meiotic cells in immature and mature cleistothecia of *A. nidulans.* In contrast to human cells, the length of telomeres throughout sexual development is the same as that found in the major vegetative cell types. These results indicate that long telomeres are probably not needed in *A. nidulans* for meiosis, but a strong regulatory mechanism is likely to exist throughout the life cycle of *A. nidulans*.

## Results

### Telomere-anchored PCR detects telomeric tracts

**Figure 1:**
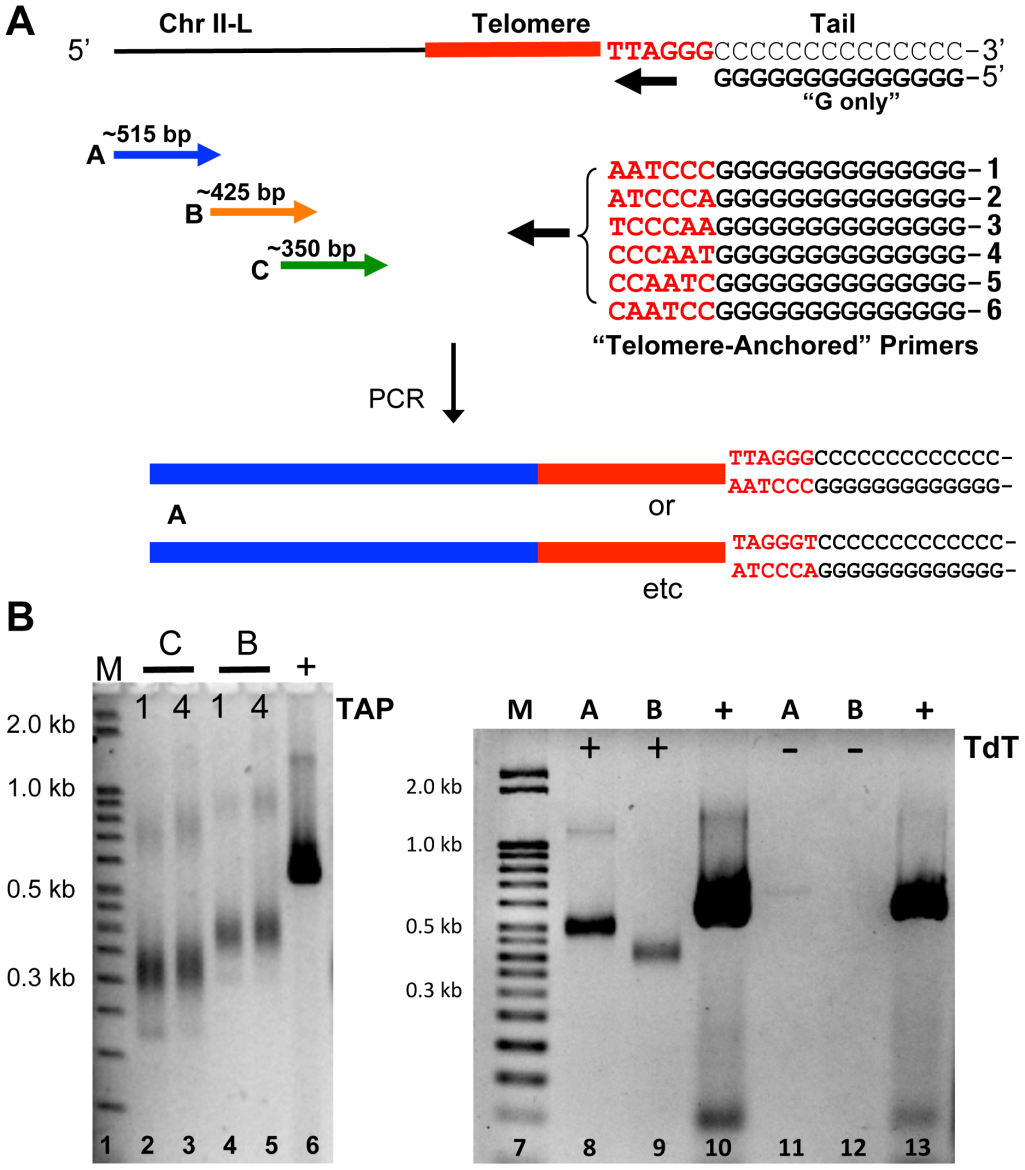
Telomere-anchored PCR assay detects telomeres in *A. nidulans.* **A.** The design of telomere-anchored PCR of C-tailed DNA using chromosome II-L. Forward primers were constructed at three different positions, labeled A, B, and C. Reverse primers were made to either a “G-only” sequence or to a G sequence that contained all six possible terminal sequences at the 3′end. **B.** PCR yielded products of expected sizes when telomere-anchored PCR primer 1 or 4 (TAP) were used with primer C (lanes 2 and 3, respectively) or primer B (lanes 4 and 5, respectively), and when telomere-anchored PCR primer 4 was used with primer A (lane 8) or primer B (lane 9). No PCR product was detected on non-tailed template without terminal transferase (TdT), using telomere-anchored PCR primer 4 and primer A (lane 11) or primer B (lane 12). The positive controls in lanes 6, 10, and 13 used primer A and a reverse primer internal to the telomere.

To determine whether PCR is a viable approach to studying telomere length in *Aspergillus nidulans,* we designed the following strategy (Fig. 1). We used three primer sequences, labeled C, B, and A, that primed in the complex chromosome-internal sequence on chromosome II-L, a unique subtelomeric region in the genome (Fig. 1A). Following a modification to the telomere PCR method that was used previously to study telomeres in *S. cerevisiae* [21], a tailing reaction was performed on *Aspergillus nidulans* hyphal genomic DNA with dCTP and terminal transferase. A 22 bp “G-only” primer was used to anneal to this C-tail, coupled with primer A (Fig. 1A). Given that the telomere tract of *A. nidulans* chromosomes such as II-L are reported to be ∼120 bp or less by Southern blot [18], and that other filamentous fungal telomere tracts are equally short [22], [23], [24], we predict that the entire PCR product would be ∼515 bp (373 bp subtelomeric region + ∼120 predicted telomeric region + 22 bp G-only primer). PCR was conducted using this C-tailed DNA as template at a variety of annealing temperatures. Although numerous products were obtained, no specific product was observed of the predicted size (data not shown). We conclude from these results that using a G-only primer is not specific enough to result in a useful length determination assay for *A. nidulans* telomere PCR.

To increase the specificity of the telomere length assay, we designed a new strategy where the reverse G primer is “anchored” to the outermost telomeric sequence. Such an anchored primer has one telomeric repeat (6 nucleotides) at the 3′ end adjacent to a G-tract (Fig. 1A), thereby predicted to anneal only to those chromosomal ends that have a telomere adjacent to the C-tail. This modification should eliminate binding of the G-only primer to a non-telomeric C-tail. Since this alteration should still require the C-tail, it is predicted to prohibit binding to inner telomeric repeats. One complication with this strategy is that it is not known which is the ultimate nucleotide of the telomeric tract of the G-rich strand in *A. nidulans*. To address this problem, we utilized all six nucleotide sequence permutations independently (Fig. 1A, telomere-anchored primers 1 through 6) with the prediction that at least one of these would correctly anchor the primer to the telomere.

We tested the ability of telomere-anchored primers (TAP) to produce the predicted size fragments with primers C, B, and A in a PCR and analyzed the results by agarose gel electrophoresis. The results showed a single, strong product for all primers tested. For example, when telomere-anchored primer 1 (Fig. 1B, lane 2) or 4 (Fig. 1B, lane 3) was coupled with forward primer C, an intense PCR product in the predicted size range (∼350 bp) was observed. When forward primer B was used independently with the same anchored primers, a larger product was observed near the expected size of ∼425 bp (Fig. 1B, lanes 4 and 5). There was also a very faint band detected at twice the size of this dominant product, most likely a PCR artifact, as it faintly appeared in many subsequent gels. When forward primer A was used independently with telomere-anchored primer 1, product in the predicted size range (∼515 bp) was observed (Fig. 1B, lane 8). Thus, the sizes of the products obtained with the three independent primers C, B, and A were all consistent with annealing of the telomere-anchored primer to the outermost telomeric sequence that had been C-tailed. Instead, if the telomere-anchored primers had annealed randomly to an inner telomeric sequence that was not immediately adjacent to the C-tail, the products would be continually shorter as PCR progressed, ultimately lacking most of the telomere. Consequently the size would be ∼ 230 bp for primer C, ∼ 310 for primer B, and ∼400 for primer A. Although there are extremely faint products occasionally observed having such sizes (e.g. Fig. 1B, lane 2, faint product at ∼235 bp), the vast majority of products is consistent with annealing of the telomere-anchored primers to the C-tailed telomeric sequence. Furthermore, when no terminal transferase was added to the tailing reaction (Fig. 1B, lanes 11 and 12), no product was obtained, indicating the PCR product is terminal transferase-dependent. Finally, if the products were due to annealing of telomere-anchored primers to the innermost telomere sequence, then the sizes of products would be unchanged with telomerase deletion (below) or with other mutants where the telomere length increases (unpublished observations), contrary to our findings.

Given the above results, we conclude that the telomeres on chromosome II-L in *A. nidulans* are specifically detected in our assay. These findings represent the first detection of a filamentous fungal telomere using a PCR assay, which we call “telomere-anchored PCR.”

### The G-rich strand telomeric terminus

When using various other means to assess telomere length, such as STELA [20] or enzymatic preparation of genomic DNA for sequence analysis, the terminal nucleotide at the 3′ end is either lost in the PCR or degraded by a nuclease. Using telomere-anchored PCR, we were now able to determine whether there was a specific nucleotide at the end of telomere II-L in *A. nidulans*, since the entire G-rich strand is intact throughout the PCR reaction.

**Figure 2:**
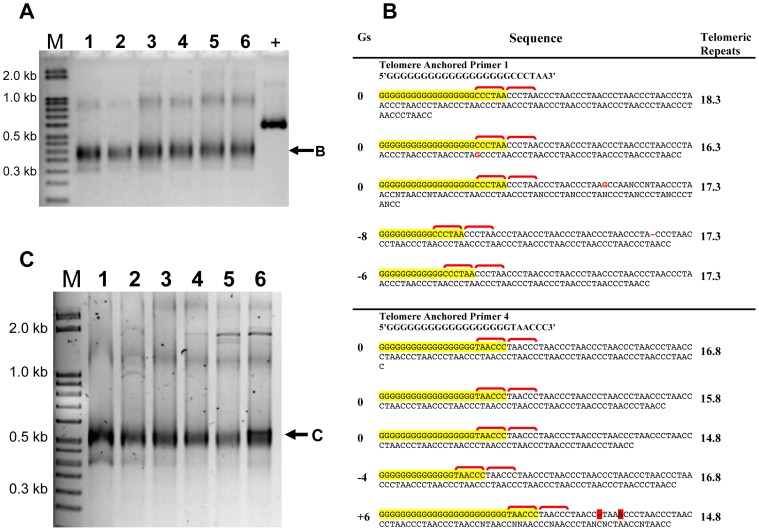
All permutations are present at the ultimate telomeric repeat. **A.** PCR products were obtained for all six telomere anchored PCR primers with no significant variation in intensity, indicating all six permutations at the ultimate sequence were amplified equally. The positive control used primers E and F. The template DNA was from an *nku-* strain. **B.** DNA sequence analysis performed on the PCR products of telomere-anchored primers 1 and 4 from panel A showed the presence of a G tract (highlighted in yellow) and the first telomere repeat (bracketed in red) as expected. The first synthesized telomeric repeat is shown in the second red bracket. The number of Gs varied from one cloned sequence to the next, and is indicated as “Gs” to the left. The number of telomeric repeats is shown at right. **C.** The template DNA was from strain GR5 having a wild-type *nkuA* gene. The telomere-anchored PCR was run using primer A coupled with the six different telomere-anchored permutation primers. PCR products were seen for all the telomere-anchored primers.

To determine if there was only one specific terminal nucleotide, we analyzed whether any single telomere-anchored primer produced a PCR product. If a particular permutation is not present at the telomere tract end, then the primer complementary to it would not anneal, and no PCR product would be obtained. Our results show that all six anchored PCR primers gave a product (Fig. 2A lanes 1–6), indicating that all permutations must be present at some level at the telomere of chromosome II-L. However, since this PCR assay is not quantitative, we cannot rule out the possibility that there is some predominance of one or more specific permutations that cannot be detected in this assay.

As validation of the sequence permutation at the terminal telomeric repeat, we obtained the precise DNA sequence data from randomly chosen clones. These clones contained PCR products made using telomere-anchored primers 1 and 4, chosen arbitrarily. DNA sequence analysis was performed from five randomly chosen independent clones for each of the two telomere-anchored PCR primers (Fig. 2B). For both telomere-anchored primers, all five clones contained the expected G tract (Fig. 2B, yellow) and the first telomere repeat (Fig. 2B, yellow plus first red bracket) found in the telomere-anchored PCR primer. In all cases, the first synthesized telomeric repeat (Fig. 2B, second red bracket), the subsequent telomeric repeats (Fig. 2B, plain), and the expected region of chromosome II-L (not shown) followed this primer sequence. Every clone contained telomeric repeats, and therefore confirmed the authenticity of the telomere-anchored PCR assay. Moreover, the permutation of the anchor sequence in the primer continued in the synthesized DNA for both telomere-anchored primers 1 and 4. If the primer did not anchor correctly, i.e. anneal to the complementary 6-nucleotide sequence, the next telomeric repeat to be synthesized would most likely be a different permutation. Thus, these sequence results indicate that 5′-TTAGGG-3′ and 5′-GGGTTA-3′ permutations, and most likely the other four permutations, are present as the ultimate telomeric repeat in the population of telomeres that was tested.

It was possible that the very terminal nucleotide may have been altered in some way, because these studies were performed for technical reasons in a *nkuA* deletion strain of *A. nidulans*. Deletion of *nkuA*, ortholog to a gene encoding Ku70, reduces nonhomologous integration [25] and has been reported to have little, if any, phenotypic consequence. An *nkuA^−^* strain, TNO2A7, is consequently used as the positive control for gene deletion experiments of many target sequences, such as the telomerase reverse transcriptase (see below). It is possible that deletion of *nkuA* may have caused variability at the ultimate nucleotide that would not normally be seen in the wild-type strain. Thus, we tested the terminal nucleotide of the telomeric tract in an essentially wild-type strain GR5, which retains the wild-type *nkuA* gene, using telomere-anchored PCR. The results indicate that the presence of *nkuA* does not lead to any specific ultimate nucleotide in the telomere (Fig. 2C), as all six telomere-anchored primers produced a discrete band as in Figure 2A. We conclude from these experiments that the ultimate nucleotide on the *A. nidulans* G-rich telomere strand is variable regardless of the presence or absence of *nkuA*. Interestingly, direct sequence determination indicates that the average telomeric tract length in the *nkuA^−^* strain, TNO2A7, has 16.5±1.1 repeats, whereas that from the wild-type *nkuA* strain GR5 has 18.4±1.5 repeats (Table 1, and also observed by Steve James, using TRF Southern blot analysis, personal communication).

**Table 1:** Cloned telomere length in wild-type and *trtA* deletion.

Strain (transformant)	Telomeric Repeats/Clone	Number of Clones	Average Length Telomere (bp)
GR5 (*nkuA*+)	18.5±1.5	3	111
*nkuA-*	16.5±1.1	10	99
*trtA-/nkuA-* (8 hours)	5.3±2.6	4	32
*trtA-/nkuA-* (16 hours)	9.3±6.9	6	56

Examination of the size of the PCR products in Figures 1C, 2A and 2C indicated they were not as distinct as expected, appearing more smeary and heterogeneous. We expected the products from one permutation primer to be homogenously sized, potentially varying by one telomeric repeat, or six nucleotides, at a time. For example, the PCR products resulting from permutation primer 1 in lane 2 (Fig. 2A) are expected to be 394 bp but may also be 388 bp or even 382 bp if some telomere tracts are slightly shorter. Likewise, lane 3 (Fig. 2A) is expected to give products of 395 bp and possibly also 389 bp and 383 bp. Even upon running this gel longer (data not shown), we were still unable to detect any sharp bands spaced six nucleotides apart. However, sequence analysis of cloned products indicated the C-tail was often variable, occasionally resulting in fewer or greater than the expected number of 18 G’s in the region corresponding to the primer (Fig. 2B, marked Gs at left). It is possible that there is also some variation in the length of the C-tail on a proportion of the molecules on the PCR product itself that might explain the smeary PCR products. Nonetheless, it is likely that such a small difference as six nucleotides is not detectable under these conditions. Regardless, the direct sequence analysis (Fig. 2B) provides evidence that all nucleotides are present at the terminus.

### Sensitivity of telomere-anchored PCR

**Figure 3:**
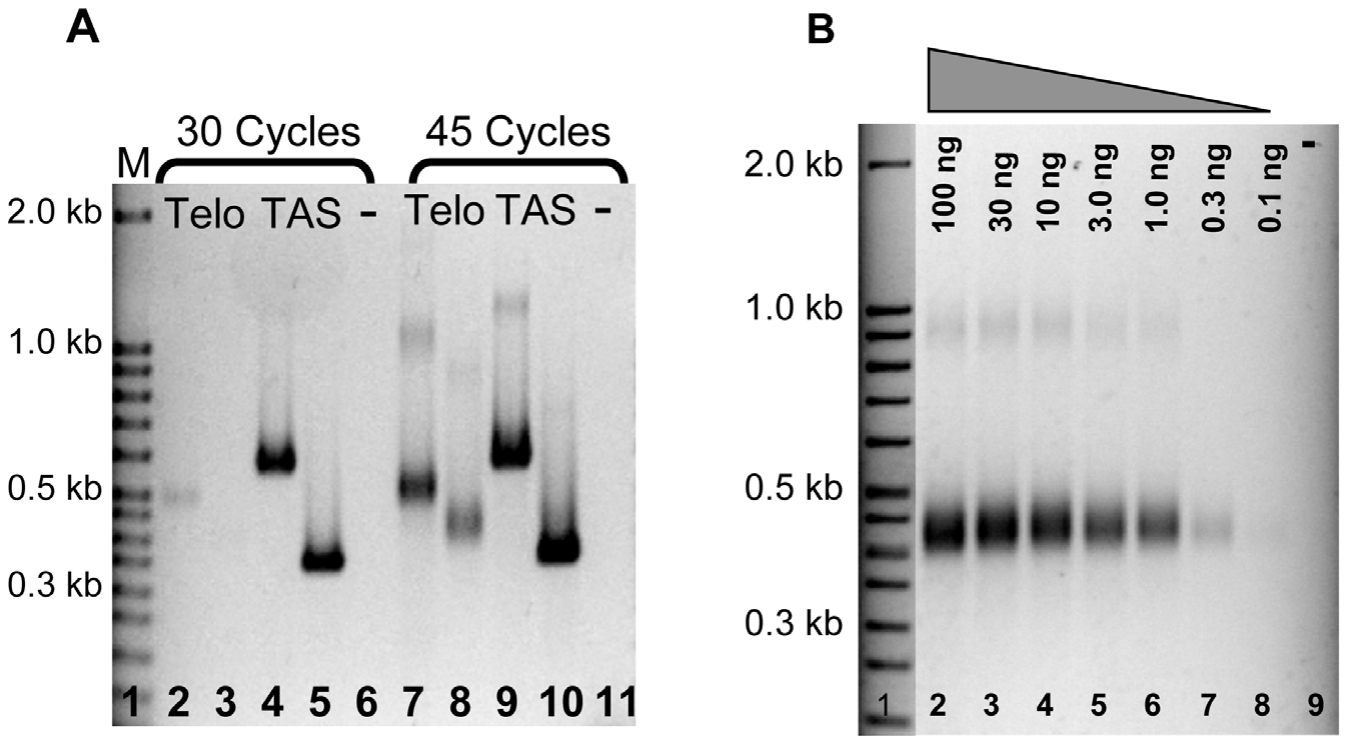
Determining the optimal conditions and sensitivity of the telomere-anchored PCR assay. **A.** PCR products were amplified for 30 cycles (lanes 2–5) or 45 cycles (lanes 7–10). Though the two telomere-associated sequences were visible at both 30 and 45 cycles (lanes 4, 5, 9, 10), the telomeric sequences were clearly seen at 45 cycles (lanes 2, 3, 7, 8). PCR was performed using either forward primer A (lanes 2 and 7) or B (lanes 3 and 8) coupled with telomere-anchored PCR primer 4. Telomere-associated sequences were used as controls in lanes 4, 5, 9, and 10. **B.** Diluted genomic DNA, ranging from 100 ng to 0.1 ng (lanes 2–8), was tailed and subjected to PCR analysis, and the intensity of PCR products was observed at a DNA concentration as low as 0.1 ng. PCR was performed using forward primer B coupled with telomere-anchored PCR primer 4.

We speculated that the telomere-anchored PCR assay is likely to be useful to assess the telomeric length of *A. nidulans* in less abundant cell types, such as ascospores within a cleistothecium, which had not been determined previously. We first needed to optimize the number of PCR cycles and determine the reaction sensitivity. To determine an acceptable number of PCR cycles to yield a good signal-to-noise ratio, we conducted telomere-anchored PCR comparing 30 to 45 cycles. Telomeric forward primers A and B (Fig. 3A, lanes 2 and 7, 3 and 8, respectively) and two different pairs of primers to telomere-associated sequences (Fig. 3A, lanes 4 and 9, 5 and 10) were used for this analysis. Results indicated that although 30 cycles were enough to detect an internal telomere-associated sequence, 45 cycles were needed to detect the telomeric tract using telomere-anchored PCR primers. Thus, 45 cycles were used for all subsequent reactions spanning the telomere.

Second, to test the sensitivity of telomere-anchored PCR to detect relatively low quantities of DNA, we subjected serially diluted DNA to tailing and telomere-anchored PCR (Fig. 3B). DNA in a concentration range 0.1–100 ng/μl was tailed and subsequently used as template for telomere-anchored PCR. Our results indicated that a telomere-anchored PCR product could be detected with as little as 300 picograms of DNA as template (Fig. 3B lane 7), which is roughly 10,000-fold less than a typical TRF Southern blot that requires about 2.5 µg DNA. Furthermore, the telomeres from the relatively sparse ascospores in *A. nidulans* can now be detected using telomere-anchored PCR. Since roughly 10,000 to 100,000 ascospores are housed within a single cleistothecium [17], at least 0.3 ng DNA can be isolated from one cleistothecium, now potentially detectable using telomere-anchored PCR.

### Telomere-anchored PCR detects ascospore telomeric DNA

To determine whether the length of the telomeric tract in *A. nidulans* is different in ascospores than in vegetative hyphae, we performed telomere-anchored PCR on DNA isolated from these less abundant sexual cells. Such a determination was not possible previously in practical terms, because Southern blot requires large quantities of DNA that cannot be readily obtained from the low number of ascospores present.

**Figure 4:**
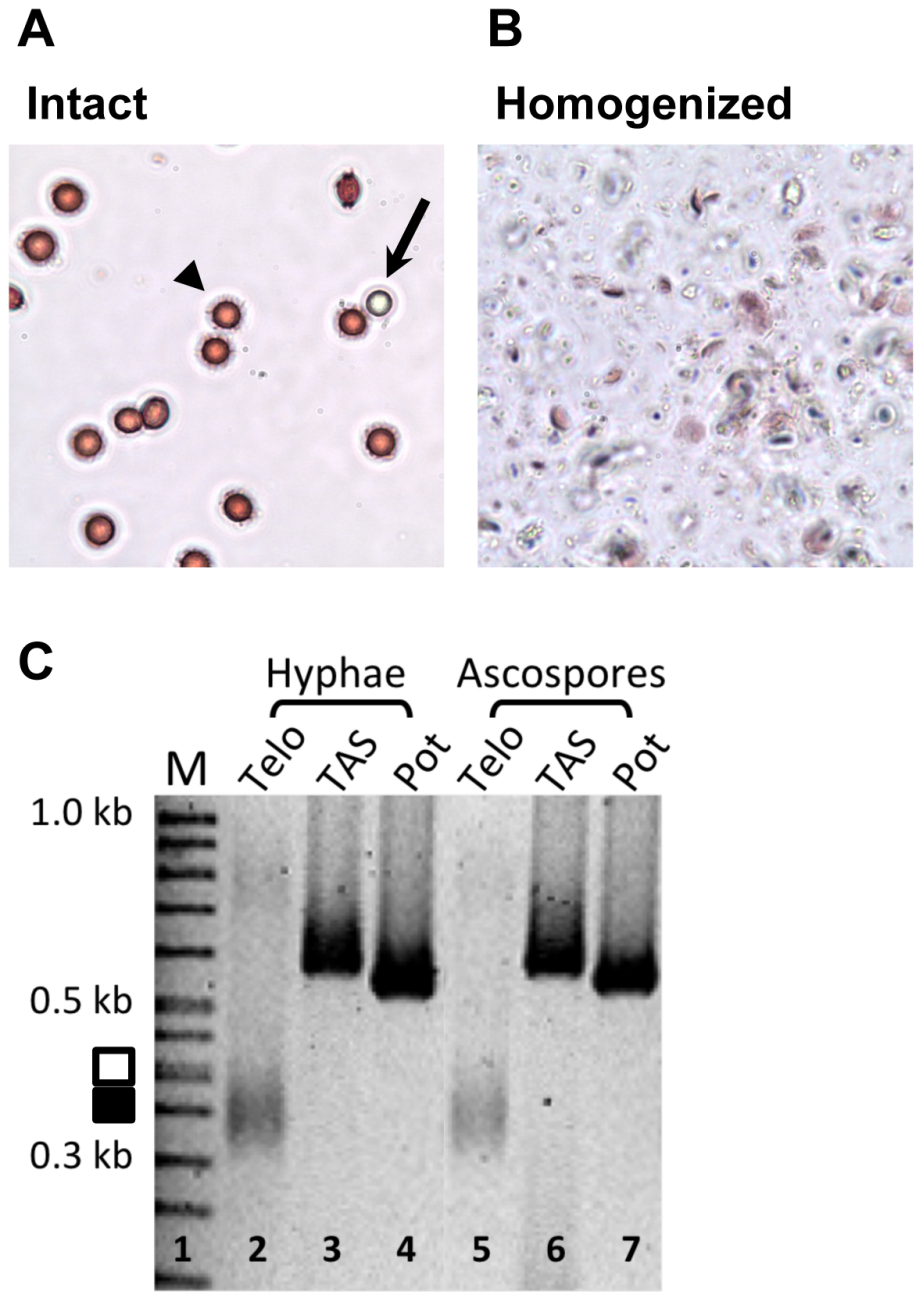
Comparable telomere lengths in *A. nidulans* hyphae and ascospores. **A.** Color microscopy of intact sexual ascospores (arrowhead) determined to be >99% of total cell concentration. The conidium (arrow) was thus relatively rare after cleaning the cleistothecium, and one is shown here for comparison. **B.** Broken ascospore lysate obtained by using a FastPrep Homogenizer. **C.** No difference was observed between *A. nidulans* hyphal and ascospore telomere length (lanes 2 and 5). Telomere PCR products for both cell types were observed at ∼350 bp. Diagram to the left corresponds to regions used for densitometry; solid black rectangle indicates majority of products and white rectangle indicates larger telomeric products. Forward and reverse primers were used to amplify telomere associated sequences (TAS, lanes 3 and 6) and the Pot1 gene (lanes 4 and 7), both located chromosome internally as controls.

Since there was no previous report of DNA isolation from ascospores in *A. nidulans*, we first established a procedure to isolate relatively pure DNA from ascospores to use as a template for PCR. Known procedures were used to clean contaminating asexual spores, or conidia, and other vegetative cells away from the surface of cleistothecia [17]. A Tris-EDTA buffer was then added, and the cleistothecia were crushed manually to release the intact ascospores. The purity of the ascospores was determined by differential cell count to be consistently at least 99% with approximately 1% or less due to conidial contamination and other negligible cell types. If conidia and ascospores are lysed at equal efficiency, this translates to a template DNA preparation that contains >99.5% ascospore DNA since the ascospores are binucleate, unlike conidia. The ascospores (Fig. 4A), were lysed on a FastPrep homogenizer (Fig. 4B), a sample was removed for microscopic analysis, and DNA was immediately isolated from the homogenate via phenol/chloroform extraction.

To determine whether the telomeres are longer in the ascospores that have completed meiosis than in vegetative hyphae, telomere-anchored PCR was conducted and the relative PCR product sizes were compared. Results indicated that there was no noticeable size difference when the ∼350 bp ascospore PCR product was compared to the hyphal PCR product on the same gel (Fig. 4C, compare lane 5 and 2, respectively). In order to determine if there was a slight difference in telomere length between the hyphal and ascospore DNA that was undetectable to the naked eye, we performed densitometry of defined regions on the gel. We determined the intensity of the products in two identical ranges of ∼50 bp for each of lanes 2 and 5: first, in the central region where the majority of the telomeric products localized and second, in ∼50 bp above the central region to determine the percentage of telomeres that were longer than those in this central region. Based on signal integration, approximately the same amount of telomeric signal was observed with the ascospore DNA ∼50 bp above the central region as in the hyphal DNA (60% versus 63%, respectively), indicating that there is virtually no increase in longer telomeres in the ascospores than in the hyphae. We also analyzed whether there was any significant difference in telomere length of ascogenous hyphae, which had nuclei in the process of meiosis, by cleaning roughly 1000 immature cleistothecia and testing a fraction of the DNA using the same conditions for telomere-anchored PCR. The results were identical to those in Fig. 4C (data not shown). A greater degree of resolution might be attained in the future if acrylamide gels were used to separate lengths of PCR products to the base pair level, rather than using agarose gels.

In summary, we found no detectable difference in the steady-state length of telomeres between ascospores and hyphae. We cannot exclude the possibility that a small fraction of the telomeres are indeed a different size but remain undetectable even with our sensitive assay, though it seems unlikely considering the nature of PCR amplification. We also cannot entirely rule out the possibility that the ∼0.5% contamination by conidial DNA template may yield the visible product and that the 99.5% of the template that originated from the ascospore yielded a product that is so heterogeneous that it cannot be detected on the gel. Nonetheless, we conclude that in this assay where we are able to identify the telomeric DNA of ascospores, this telomeric DNA appears the same length as that of vegetatively growing hyphae, approximately 110 bp.

### Telomere-anchored PCR detects small length decreases of telomeric tracts in telomerase mutants

A mutation in *A. nidulans* that results in length change of the overall telomeric tract has not been reported, so it was not yet possible to test such changes using our telomere-anchored PCR approach. It was therefore desirable to first construct a mutant that would result in a predictable effect on telomere length, such as the TERT gene, the protein product of which is necessary for the maintenance of telomeres. In a variety of organisms, genetic deletion of TERT results in shorter telomeres and usually cell death [26].

**Figure 5:**
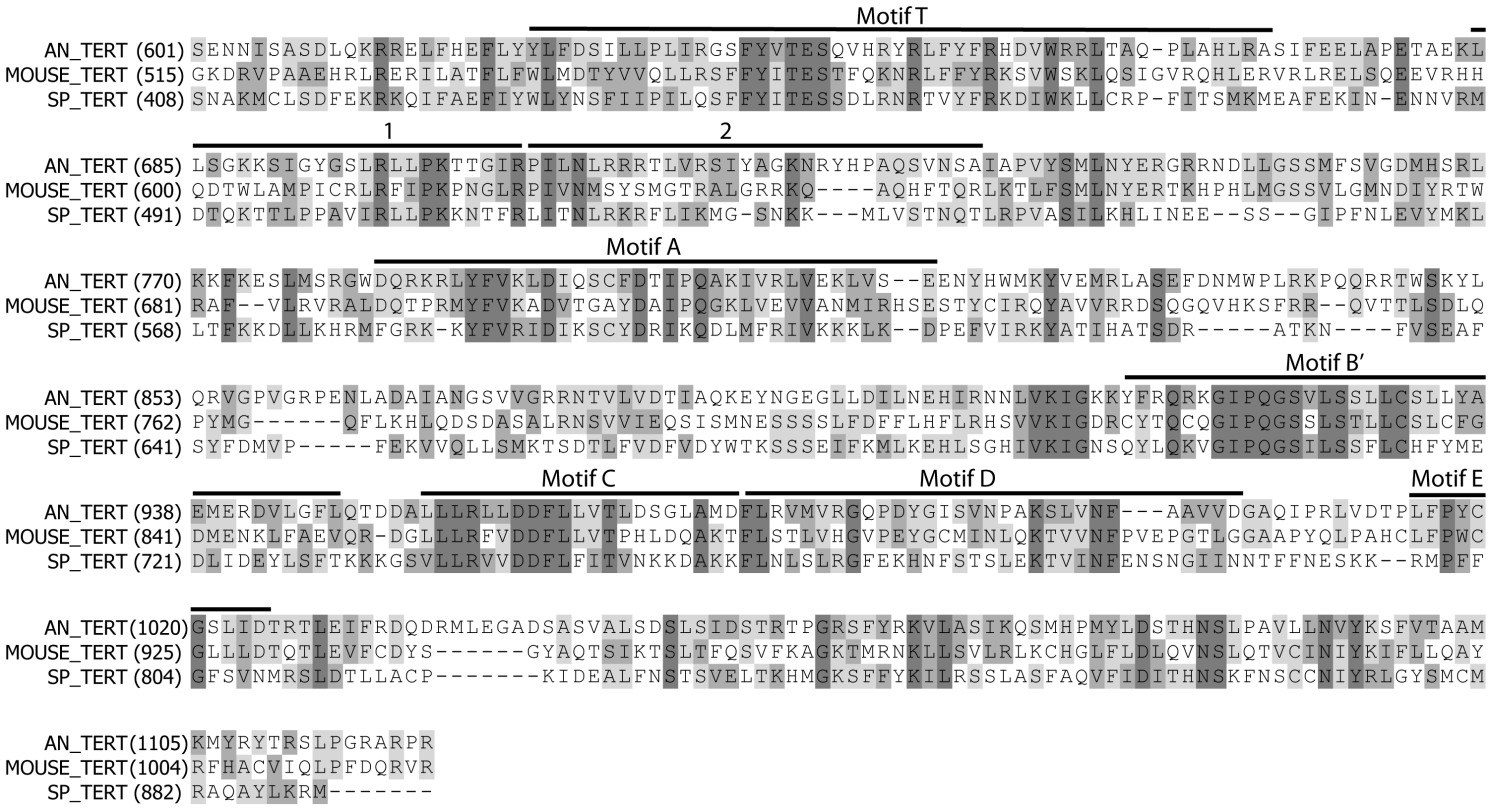
Alignment of an *A. nidulans* ORF to known TERT motifs. The ANID_03753.1 predicted protein sequence (AN_TERT) was aligned to the TERT sequence from mouse (MOUSE_TERT, accession number O70372) and *S. pombe* (SP_TERT, accession number O13339) using the Align program in Vector NTI (Invitrogen). The region of the alignment including AN_TERT residues 601 through 1120 were selected and are shown. The conserved telomerase reverse transcriptase motifs (Motif T, 1, 2, Motif A, Motif B, Motif C, Motif D and Motif E) are indicated above the aligned sequences. Residues identical in all three sequences are in shown with dark shading, whereas sequences that are similar or identical in only two sequences are shown in lighter shading.

To identify the *A. nidulans* telomerase reverse transcriptase gene, we searched the *A. nidulans* genome using the *S. pombe* telomerase reverse transcriptase catalytic subunit (accession number O13339) as the query. This BLAST search identified a single coding sequence designated ANID_03753.1 (*A. nidulans* sequencing project, Broad Institute of MIT and Harvard, http://www.broad.mit.edu). An alignment of the carboxyl-terminal regions of the predicted *A. nidulans* sequence with the TERT sequence of fission yeast and mice illustrates that the *A. nidulans* sequence contains the seven motifs found in all reverse transcriptases and also the T motif, which is specific to TERT proteins (Fig. 5) [27]. Comparison of similarity using protein sequence alignments of the ANID_03753.1 predicted product to known TERT proteins strongly suggests that ANID_03753.1 is the *A. nidulans* TERT gene, which we now will refer to as *trtA*.

**Figure 6:**
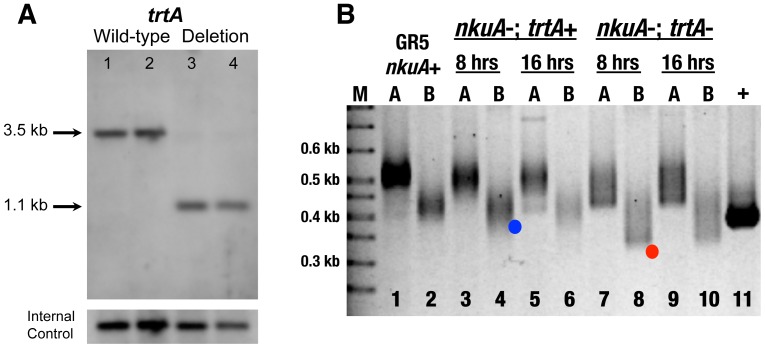
Telomerase mutants showed dramatic shortening of telomeres compared to wild-type as assayed by telomere-anchored PCR. A. Genomic DNA was isolated from wild-type strains (GR5 in lane 1, TN02A7 in lane 2) and two putative *trtA* deletion mutants (TDP3-2 in lane 3, TDP3-32 in lane 4), digested with EcoRI and analyzed by Southern blot using PCR product A as probe. B. Telomere-anchored PCR was performed on genomic DNA isolated from wild-type and *trtA* deletion mutants with either forward primer A or B coupled with telomere-anchored PCR primer 4. At both eight hours and sixteen hours after transformation, *trtA* deletion mutants show significantly shorter telomeres (lanes 7-10, red dot in lane 8) than wild-type (lanes 1-2) and *nkuA* deletion mutant (lanes 3-6, blue dot in lane 4).

The *trtA* deletion mutant was created by one step gene replacement using the heterokaryon rescue technique [28]. Multiple independent transformants were recovered that displayed a phenotype consistent with the loss of an essential gene. Two transformants (TDP3-2 and TDP3-32) were chosen for further analysis. TDP3-2 and TDP3-32 produced spores that could germinate on selective medium but halted growth before forming visible colonies (data not shown). A report on the detailed cytological analysis of this mutant is beyond the scope of this report. To confirm the identity of the *trtA* deletion mutant, genomic DNA from germinated conidia of wild-type and the TDP transformants was analyzed by Southern blot (Fig. 6A). A probe specific to *trtA* identified a predicted 3.5 kb fragment in wild-type strains (Fig. 6A, lanes 1 and 2) and a predicted 1.1.kb fragment in the mutants (Fig. 6A, lanes 3 and 4). PCR analysis (data not shown) confirmed the Southern blot results. Based on this analysis, we conclude that TDP3-2 and TDP3-32 are *trtA* deletion mutants.

To determine whether our telomere-anchored PCR assay could detect a change in the telomere length predicted by the *trtA* deletion, we tailed genomic DNA from TDP3-2 and TDP3-32 germlings and carried out telomere-anchored PCR with either forward primer A or B coupled with telomere-anchored primer 4, chosen arbitrarily. Our results showed a dramatic telomere length decrease in telomerase deficient cells (Fig. 6B), although the distribution of the telomeres was heterogeneous in length. In as little as 8 hours growth, a large fraction of the *trtA^−^ nkuA^−^* telomeres had approximately six repeats remaining, as calculated from the size of the leading edge of the PCR product compared to the *trtA^+^ nkuA^−^* telomeres that had approximately the wild-type number of 16 repeats (Fig. 6B, compare red dot in lane 8 with blue dot in control *trtA^+^ nkuA^−^* lane 4). No further loss of telomere tract length in *trtA^−^ nkuA^−^* cells was observed at 16 hours in the remaining telomeres. The entire heterogeneous telomeric DNA was excised from lanes 7 and 9 of the agarose gel, telomeres were cloned, and DNA was sent for sequence analysis. Results indicated the shortest telomere had 3 repeats, or roughly 20 bp (Table 1), present in the sample from 8-hour growth. The average telomere length was shorter in the 8-hour sample than in the 16-hour sample, which may be due to growth of *trtA^+^/trtA^−^* diploids with increased time. Diploids can be produced along with the *trtA^−^* haploid conidia at a frequency of approximately 10*^−^* ^6^ by such heterokaryons (data not shown). Though rare, these diploids outcompete the sick haploids and their nuclei increase in representation as the culture time increases.

In summary, we have verified using *in vivo* mutagenesis that the open reading frame identified in silico is indeed the telomerase reverse transcriptase. This is the first time that deletion of the telomerase reverse transcriptase gene has been experimentally demonstrated to result in shorter telomeres in filamentous fungi. Moreover, our telomere-anchored PCR assay is sensitive enough to detect small decreases, for instance from 100 bp to 50 bp in the overall telomere tract length. This assay will be useful for future determination of telomere size in *Aspergillus nidulans*.

## Discussion

This report represents the first identification of filamentous fungal telomeres using a PCR approach. This quick and relatively inexpensive technique, which we term telomere-anchored PCR, was used to determine the telomere length of chromosome II-L of *Aspergillus nidulans* cells. Furthermore, we determined the telomere length of this chromosome arm in the sexual cells in *A. nidulans*, the first in any filamentous fungus. These studies extend what was previously identified as exceptionally short telomeres in filamentous fungi [18], now indicating that the entire life cycle, including the sexual stage, has telomeres that are approximately 110 bp throughout the stages of this fungus' relatively complex life cycle. Furthermore, this report provides genetic verification of the putative gene sequence for telomerase reverse transcriptase in *A. nidulans* (here called *trtA*), where we observed a decrease in telomere length during growth of cells lacking the *trtA* gene. All of these findings, coupled with the relative ease of molecular genetics in *A. nidulans*, contribute to the establishment of this filamentous fungus as a new model organism for studying telomere length regulation.

The telomere-anchored PCR assay developed here might be utilized in a wide variety of fungi, yeasts, or potentially other organisms such as vertebrates. The requirements to develop such an assay in another model organism are knowledge of the subtelomeric sequence to develop a PCR primer, a telomeric repeat sequence that is invariant, and potentially the relatively shorter telomeres found in most fungi and lower eukaryotes. Preliminary results suggest telomere-anchored PCR can be used to determine the telomere length of *Apergillus oryzae* (unpublished), and the length is consistent with that found by Southern blot analysis [24]. Telomere-anchored PCR may be useful for determining the telomere length of other filamentous fungi, such as *Neurospora crassa* [22], or in species of yeast that have invariant telomere repeats, such as members of *Kluveromyces* or *Candida* [29], [30]. It may also be useful to establish the maximum telomere length this method can detect in plants or vertebrates.

In developing a PCR assay for telomere length, one consideration was to copy the entire G-rich strand so that future studies might address the G-rich overhang. Thus, we attempted an assay that was similar to telomere PCR used in yeast [21], which can also determine the entire length of the G-rich strand. We did not achieve the necessary specificity, however, until we devised a strategy that utilized an anchor at the 3′ end of the primer to the terminal six nucleotides of the 3′ end of the G-rich overhang. Therefore, telomere-anchored PCR might be able to eliminate any non-specificity that might occur in other organisms. Various PCR methods have been used in other organisms, but in these methods the terminal nucleotides of the 3′ G-rich strand are not copied [19], [29]. For example, the PETRA and STELA PCR assays used in plants [30] and humans [20], respectively, rely on annealing of a primer to the 3′ G-rich overhang, but they cannot indicate the absolute length of the G-rich strand as it is not fully copied. Our results suggest that the G-rich strand ends in all six possible permutations (TTAGGG-3′, TAGGGT-3′, AGGGTT-3′, etc) but further analysis of the terminal nucleotides of the G-rich strand will be interesting. In ciliates, predominantly a single terminal nucleotide at the G-rich strand exists, and in humans, ∼40% of the telomeres end in a single nucleotide [31], [32], [33]. The heterogeneity suggested here is similar to *S. cerevisiae*, which has no apparent nucleotide predominance at the G-rich strand [21].

Many filamentous fungi have TTAGGG repeats [14], and at least *Aspergillus oryzae* [24], *Neurospora crassus* [22], and *Magnaporthe oryzae* [23] contain short, homogenous telomeres like those of *A. nidulans*. Interestingly, the telomere repeat of *A. oryzae* (TTAGGGTCAACA) has six additional nucleotides, yet the overall telomere tract length is ∼120 bp [24]. Thus, even though the telomeric repeat is twice as long in *A. oryzae*, the overall tract length is virtually the same between *A. oryzae* and *A. nidulans*. This observation suggests that the length of the telomere tract rather than the number of repeats is conserved among filamentous fungi. Moreover, there may be some fundamental mechanism that keeps telomeres in filamentous fungi short and homogenous. Although loss of a few telomeric repeats can be tolerated in an organism having longer telomeres, such a loss might be devastating in *Aspergillus nidulans*.

This emphasizes a fundamental question regarding telomere length in *A. nidulans* and potentially in other filamentous fungi: how does telomere length stay relatively homogenous, barely differing by more than one repeat on a tract of 18 repeats, during all stages of the fungal life cycle? In other words, the telomere tract length is undeviating, potentially allowing telomerase and other telomere-associated proteins to add on just enough telomeric repeats. It is possible that telomerase adds on more telomeric repeats than is needed but are then degraded. Since our assay detects the ultimate nucleotide of the G-rich strand and therefore can detect lengthening of this strand by telomerase, such a scenario seems unlikely. We did not detect telomere lengthening in any stage of the organism's life cycle. Even in sexual cells that just went through meiosis, no change in telomere length was observed. Therefore, not only is telomere lengthening not essential for any aspect of meiosis that can be detected in *A. nidulans*, the telomere length appears unchanged during all stages of mitotic and meiotic division.

It would be intriguing to uncover how telomerase and other proteins are regulated to maintain the short telomere length in *A. nidulans* and other filamentous fungi. The formation of a t-loop, [34], [35], which forms a protective cap by invasion of the G-rich overhang into the adjacent double stranded telomere and telomere-associated proteins, may not be possible in these fungi. Although the t-loop has been shown to exist in a wide variety of organisms, including yeast [36], it is unclear whether a t-loop as small as 110 bp could be observed with current methodologies. Of the two nuclei of a single-celled ciliated protozoan, a t-loop has been observed to cap the several-kilobase pair telomere of the chromosomes in the micronucleus but has not been observed in the 36-nucleotide telomeres that flank gene-sized DNA molecules in the macronucleus [37]. Interestingly, a Pot1 ortholog has been identified in *A. nidulans* [38]. Pot1 protein protects short telomeres where a t-loop may not be formed, by binding to the G-rich telomeric overhang [39]. Coupled with the ease of the assay demonstrated here, which detects the G-rich strand of the telomere, it is now possible to examine the role of the Pot1 protein in the capping of telomeres in this fungus.

## Materials and Methods

### Growth of strains and cell type isolation

Strain A4 is Glasgow wild-type (veA+); Strain TN02A7 is pyrG89; pyroA4;nkuA::argB; riboB2 [25]; strain GR5 is pyrG89;wA3;pyroA4. Liquid cultures of hyphae were grown by inoculating spores at a final concentration of 3.0×10∧6 spores/ml in 50 ml complete media [40]. Most cultures were incubated at 37° with shaking at 120 RPM overnight. Hyphae were isolated from the media by vacuum filtration through Miracloth (Calbiochem). For sexual cells, mature cleistothecia were obtained by incubating strain A4 on complete media plates unsealed for 3 days at 37°, then covered in Parafilm M and incubated for three additional days, whereas immature cleistothecia were taken from plates incubated for 3 days unsealed or for 3 days unsealed plus one additional day of incubation. Mature and immature cleistothecia were cleaned of asexual cells by rolling on a 3% agar Petri plate.

### Isolating genomic DNA

Hyphal DNA was first isolated by adding either a small amount of sand to 50 mg lyophilized hyphae or liquid nitrogen to 100 mg of wet hyphae and crushing with mortar and pestle. However, the following new method was preferred, given the ease of DNA recovery and no obvious differences in subsequent PCR assay results. In this method, 50 mM Tris and 100 mM EDTA were added to filtered hyphae or cleaned cleistothecia and placed into a tube with Lysing Matrix C beads (MP Biomedicals) and homogenized in a FastPrep™24 or a Precellys 24 tissue homogenizer three times at 5500 rpm for 20 s for the latter instrument, placing on ice for 1.5 min in between each cycle. DNA was extracted with an equal volume of Phenol/Chloroform/Isoamyl Alcohol. DNA was isolated using a Qiagen DNeasy Plant Mini Kit (Qiagen) following manufacturer's recommendations or later a GeneClean Turbo Kit (qbiogene). The DNA was quantified by running samples alongside a High DNA Mass ladder (Invitrogen) or by a BioSpec-Nano spectrophotometry (Shimadzu). For cleistothecia DNA isolation, 250 µl of 50 mM TRIS and 100 mM EDTA were added to 300 mature or 1000 immature cleistothecia and crushed with a glass rod. For mature cleistothecia, the number of ascospores and conidia was counted in a hemacytometer. The sample was added to Matrix C beads and procedure described above was followed.

### Microscopy

Photomicrographs of mature cleistothecia having been crushed to release ascospores, or crushed and homogenized on a FastPrep™, were taken using a Nikon microscope at 1000× magnification with phase optics and a Spot Insight™ camera. To determine the general timing of meiosis with a cleistothecium, immature cleistothecia were gently smashed under a coverslip and assessed as having only ascogenous hyphae with no visible signs of ascus development.

### Telomere-anchored PCR

Approximately 200 ng *A. nidulans* DNA was tailed in 50 mM potassium acetate, 20 mM Tris acetate, 10 mM magnesium acetate, 1 mM dithiothreitol, 0.25 mM cobalt chloride, 0.1 mM dCTP, 0.1 U Terminal Transferase (New England Biolabs) at 37°C for 30 minutes and heat denatured at 70°C for 10 minutes. Non-tailed DNA was prepared the same as tailing with the exception that H_2_0 replaced terminal transferase. PCRs with tailed or non-tailed DNA were performed in 25 µl with 1 µM telomeric primer, 1 µM subtelomeric primer, approximately 10 ng C-tailed genomic DNA, and 25 µl JumpStart REDTaq ReadyMix Reaction Mix (Sigma). The PCRs were started for 94°C for 2 minutes, then run for a total of 45 cycles (30 cycles were used in some cases), using the following cycle conditions: 94°C for 30 seconds, 64°C for 30 seconds or 60°C for Pot1 control, and 72°C for 1 minute. Reactions were completed at 72°C for 5 minutes, 4°C hold, unless otherwise indicated.

Primers used for telomere-anchored PCR (Eurofins MWG Operon):

Telomeric primer permutation 1, 5′-(G)_18_CCCTAA-3′; Telomeric primer permutation 2, 5′-(G)_18_ACCCTA-3′; Telomeric primer permutation 3, 5′-(G)_18_AACCCT-3′; Telomeric primer permutation 4, 5′-(G)_18_TAACCC-3′; Telomeric primer permutation 5, 5′-(G)_18_CTAACC-3′; Telomeric primer permutation 6, 5′-(G)_18_CCTAAC-3′; G-only primer, 5′-(G)_22_-3′; OutermostRevPrimCh-2 (Primer A), 5′-CAATTTGTTCAT AGCCAGCTGATACGGATGGGCC-3′; OutermostForPrimCh-2 (Primer D), 5′-GGGCGTCAAGGTTGTCAAAAAGGTAACGGTGTTC-3′; LingForwardTelPrimer (Primer B), 5′-GGTCAAGTTCCCCTAGCAGCGTGGGCCATCAGTATCTACATGTAC-3′; LingOuterForPrim-Ch2 (Primer E), 5′-GTACATGTAGATACTGATGGCCCACGCTGCTAGGGGAACTTGACC-3′; LingOuterRevPrim-Ch2 (Primer F), 5′-CTCGATACCGGCACTCTGAATAGCAGCGACCCTCTGACATAAC-3′; Chr2LongforwardPrim1 (Primer C), 5′-CAAAGCATTCACTACCTGGTCTATGACACCCC-3′; Outer ForwardP nimU (For Pot1 primer), 5′-GATACAGTGCTGACTGGCCTC-3′; Outer ReverseP nimU (Rev Pot1 primer), 5′-GATGCTCATACGACCGACTGG-3′.

### Cloning and DNA sequence analysis

Gel excision and DNA extraction were conducted following the QIAquick Gel Extraction Kit protocol (Qiagen), and cloning was carried out following the TOPO TA Cloning Kit for Sequencing protocol (Invitrogen) in One Shot Chemically Competent *E.coli.* Plasmid DNA was isolated using the QIAprep Spin Miniprep Kit (Qiagen) and sent to the University of Chicago, CRC-DNA Sequencing Facility for sequence analysis.

### Gel electrophoresis and densitometry

Typically agarose gel electrophoresis was performed using 2.5% agarose and 1× TBE Buffer [41], DNA was stained with ethidium bromide, and analyzed using a Bio-Rad VersaDoc Imaging System and Quantity One analysis software. Densitometry measurements were performed by drawing a central rectangle corresponding to ∼50 bp region of the gel where the majority of the PCR products localized, and then drawing the same size rectangle above central rectangle. The volume of the signal was determined within each rectangle after subtracting the background signal.

### Creation of *trtA* deletion mutants

The *trtA* deletion mutant was created by transformation of strain TN02A7 with a knockout construct designed to replace *trtA* with the Af *pyrG* selectable marker [25]. The knockout construct, designed to delete from 90 bp before the predicted ATG start codon through 100 bp after the predicted TGA stop codon, was created by a fusion PCR [42] using three PCR products A, B, and C as template and primers P2 and P5. PCR product A was generated using genomic DNA as template and primers P1 and P3. Product B was generated using pXDRF4 [42] as template and primers pyrG-for and pyrG-rev. Product C was generated using genomic DNA as template and primers P4 and P6.

The heterokaryon rescue technique [28] of strain TN02A7 was used. Transformation with the ANID_3753.1 knockout construct yielded heterokaryotic transformants that were consistent with deletion of an essential gene [28], which produced condia, and provided sufficient DNA for analysis when germinated in medium lacking uracil. PCR and Southern blotting analysis confirmed deletion of ANID_3753.1. For PCR, primers P1 and P6 were used (Expand Long Template PCR system by Roche), which generated a 6123 bp band in wild type and a 4044 bp band in the deletion mutants. For Southern blot analysis, PCR product A used to generate the deletion construct was used as probe.

Primers used for telomerase knockout construction:

P1, 5′-TCCGCACCCACCAGTCACCA-3′; P2, 5′-TGTGGGCTTGAATGTGCAGG-3′; P3, 5′-GTTCCACGATGGTGTAGTCCTACAGGGGTGGTCGCTGAACG-3′; P4, 5′-TCAGTGCCTCCTCTCAGACAGAGGGAGCCAAGGGGTCGGTA-3′; P5, 5′-AGCGGTGCACAGTGCGTTA-3′; P6, 5′-TGGCCCCAACCACGCTGTAC-3′; pyrG forward, 5′-AGGACTACACCATCGTGGAACAGT-3′; pyrG reverse, 5′-CTGTCTGAGAGGAGGCACTGATGC-3′.
